# Preparing Physicians for Tomorrow’s Practice: Contemporary Practice Competencies as a Framework for Technology-Driven Change

**DOI:** 10.2196/90637

**Published:** 2026-04-17

**Authors:** Joseph E Safdieh, June Chan, Domenick Falcone, Erika L Abramson

**Affiliations:** 1 Weill Cornell Medicine New York, NY United States

**Keywords:** medical education, artificial intelligence, competencies, undergraduate, curriculum, contemporary practice competencies

## Abstract

Medical education has long relied on stable, high-level program objectives to articulate the outcomes of undergraduate medical training. These objectives have served an essential role in defining professional identity, guiding curricular design, and ensuring accountability. However, the pace of contemporary clinical change increasingly exceeds the capacity of static curricular structures to adapt. Expectations for how physicians practice, communicate, and exercise judgment, particularly in technology-mediated care environments, are evolving faster than traditional curricular governance mechanisms can accommodate. Recent accreditation developments, including proposed changes to the organization of required content domains, signal a broader inflection point: medical schools are being challenged to move beyond additive, topic-based approaches to curriculum design toward more durable and adaptive frameworks for preparing physicians for practice. This viewpoint argues that many of the tensions currently facing medical education reflect an overreliance on problem lists and topical coverage models that were designed for a slower era of clinical evolution. As new priorities emerge, ranging from artificial intelligence and data-driven decision support to prevention, climate health, and changing patient expectations, curricula risk becoming increasingly dense, fragmented, and difficult to govern. Simply elevating each new priority to the level of a program objective undermines the coherence and longevity of objective frameworks and politicizes curricular decision-making. To address this challenge, we propose contemporary practice competencies (CPCs) as a complementary, middle-layer construct positioned between medical educational program objectives and course- or clerkship-level objectives. CPCs are dynamic, revisable competencies designed to translate stable educational aims into forward-facing clinical capabilities, particularly in domains where technology and data-driven tools are reshaping clinical reasoning. Using artificial intelligence as a primary test case, we illustrate how CPCs enable longitudinal, assessable development of emerging practice capabilities without repeatedly restructuring high-level objectives. By providing a transparent and adaptable mechanism for curricular evolution, CPCs offer a practical approach to aligning medical education with the accelerating realities of contemporary clinical practice.

## Overview

Medical school curricula have always evolved in response to forces beyond the lecture hall, such as emerging diseases, shifting demographics, scientific discoveries, and emerging therapeutics. What has changed is the pace of clinical evolution. Expectations for how physicians practice, communicate, and earn trust are shifting faster than the mechanisms used to guide curricular change, which often remain reliant on static content categories, infrequently revised objectives, and additive approaches to new priorities. Recent accreditation developments, including the removal of “societal problems” as a discrete curricular category by the Liaison Committee on Medical Education (LCME) [1], mark an important inflection point, challenging medical schools to move beyond continually expanding catalogs of required topics designed to react to problems.

One useful way to interpret this moment is as a shift from a problem-list paradigm to a practice-readiness paradigm. Historically, curricular requirements have been periodically expressed through topical expectations, what might be called “lists of importance.” Many schools responded by creating explicit coverage maps, adding sessions, and, at times, promoting specific content areas into high-level educational program objectives. Over time, this approach becomes vulnerable to curricular inflation. New priorities compete for permanent placement, the curriculum grows denser, more fragmented, and less teachable. Educational leaders and learners recognize that overcrowded curriculum undermines deep learning, invites superficial coverage, and can compound stress and burnout [2].

The elimination or reorganization of content expectations previously grouped under “societal problems” offers an opportunity to recalibrate. This is not an argument that social and structural drivers of health no longer matter but a question of framing. A “societal problems” construct is inherently reactive, implying that curricula should follow emerging pathologies. Contemporary practice increasingly demands something different: the ability to anticipate, prevent, and redesign care. The aim of this viewpoint is to propose a conceptual framework, contemporary practice competencies (CPCs), to support the integration of evolving domains of clinical practice into medical education while preserving the stability of high-level program objectives.

This tension is particularly evident in areas now central to contemporary practice but awkward to incorporate as discrete topics, including artificial intelligence (AI) in clinical decision-making [3], climate change, nutrition in the prevention and management of chronic disease, and communication in an era of erosion of public trust in science. Among these, AI offers a particularly clear illustration of why traditional curricular structures struggle to keep pace with practice evolution.

## Medical Education Program Objectives Should Remain Thematic, Not Topical

LCME-accredited medical schools already have a stable set of Medical Education Program Objectives (EPOs) that are intentionally broad, thematic, and durable. EPOs describe the physician the program aims to produce. Their strength lies in their longevity. Their limitation is that they can become too abstract to reliably drive curricular design when practice shifts rapidly.

This is where schools face a recurring temptation: if AI is important, make it an EPO; if nutrition is important, make it an EPO; if prevention is important, make it an EPO. But high-level program objectives are meant to describe enduring capacities, not the shifting topical priorities of a particular decade. Once objectives become topical, governance becomes politicized as every emerging priority becomes a candidate for canonization, and every specialty’s “pet project” risks being framed as universally required. The result is not curricular coherence; it is curricular lobbying.

As a recent example, the integration of nutrition into medical education highlights this challenge. Recent consensus efforts have identified a broad set of nutrition competencies spanning foundational knowledge, clinical assessment, patient communication, public health, and interprofessional care. These competencies cut across multiple domains of physician performance and are expected to be developed longitudinally across training rather than confined to a single course or objective. As a result, nutrition resists clean alignment with any single EPO. Mapping it to one domain risks fragmentation, while distributing it across many risks dilution and loss of accountability. This illustrates a broader structural limitation: some contemporary practice capabilities are inherently cross-cutting and longitudinal and therefore difficult to operationalize within static, high-level objective frameworks alone [4].

## A Missing Middle Layer: CPCs

We propose a complementary construct: CPCs, a dynamic, revisable set of competencies designed to sit between enduring EPOs and course- and clerkship-level objectives. CPCs are not replacements for EPOs. They function as an implementation layer, translating stable, thematic outcomes into forward-facing clinical capabilities that must be developed intentionally over time. Conceptually, CPCs serve as an organizing framework for evolving clinical practice priorities within established EPOs.

CPCs are defined by 4 design principles. First, they focus on capabilities physicians need in current and emerging clinical practice, rather than on static content areas. Second, they are linked to existing EPOs, ensuring that new domains are integrated without altering the overall structure of the objective framework. Third, CPCs are developed across the curriculum over time, rather than introduced as isolated topics or single sessions. Finally, CPCs are assessed through observable clinical work, using existing approaches such as entrustable activities and workplace-based assessments rather than relying solely on content coverage.

CPCs align with the logic of entrustable professional activities (EPAs), which were introduced to bridge the gap between abstract competency domains and the real work of clinical practice [5]. While EPAs are often framed around discrete units of clinical work that can be observed and assessed in context, the underlying insight is broader: learners and faculty need a practical, observable language for readiness that can be mapped back to higher-order outcomes. However, EPAs are generally stable and task-based and are not designed to accommodate rapidly evolving, cross-cutting domains of practice that must be integrated longitudinally across the curriculum. CPCs extend this logic by providing a dynamic, intermediate layer that translates enduring program objectives into evolving, cross-cutting capabilities (Table 1). In this way, CPCs provide a structured mechanism for incorporating domains such as nutrition, AI, and climate health across disciplines without elevating each to a distinct EPO.

**Table 1:** Conceptual relationship between educational program objectives, contemporary practice competencies, and entrustable professional activities, highlighting how CPCs function as an intermediate layer linking stable program objectives to observable clinical work.

Level	Purpose	Characteristics	Example
Education program objectives (EPOs)	Define the physician the program aims to produce	Broad, thematic, stable over time	Perform both a focused and comprehensive history and physical examination, develop diagnostic hypotheses, order and evaluate diagnostic tests, and formulate an appropriate plan of care.
Contemporary practice competencies	Translate EPOs into evolving, cross-cutting capabilities	Dynamic, revisable, longitudinal, linked to multiple EPOs	Integrate artificial intelligence–supported clinical information into diagnostic reasoning and patient-centered care planning across clinical contexts.
Entrustable professional activities	Define units of observable clinical work	Task-based, context-specific, assessable in practice	Prioritize a differential diagnosis following a clinical encounter.

A key distinction is that EPAs are designed to organize clinical work, not to define evolving domains of practice. While EPAs can incorporate new elements such as the use of AI within a diagnostic encounter, they do not provide a mechanism for identifying, updating, and longitudinally integrating new capabilities across the curriculum as practice evolves. In contrast, CPCs are explicitly designed to define these evolving domains and to guide their development over time. In this way, CPCs complement EPAs rather than duplicate them: CPCs define what capabilities are needed, while EPAs define how those capabilities are enacted in specific clinical tasks.

For example, in the domain of AI, a CPC may define the expectation that learners develop the ability to integrate AI-supported clinical information into diagnostic reasoning and patient-centered care across clinical contexts. This is not a single observable task, but a longitudinal capability that informs multiple EPAs, such as prioritizing a differential diagnosis or developing a management plan. In this way, CPCs define what should be developed across the curriculum, while EPAs and associated assessment approaches define how that capability is demonstrated and evaluated in specific clinical contexts.

## Why AI Is an Ideal Test Case

AI highlights the limitations of topic-based curricular design and the advantages of a CPC framework. If elevated to an EPO, AI would immediately compete with genomics, climate health, nutrition, and other emerging priorities, destabilizing the objective layer. More fundamentally, AI evolves too rapidly to be governed as a static content domain. Models change, tools are updated, and clinical applications expand faster than curricular approval cycles can accommodate.

Framed as a CPC, AI becomes a longitudinal capability rather than a discrete topic. Learners can be expected to develop the ability to critically evaluate AI-supported clinical recommendations, recognize limitations and sources of bias, integrate algorithmic outputs into clinical reasoning, communicate uncertainty with patients, and maintain professional judgment in technology-mediated environments. These capabilities can be scaffolded across the curriculum and assessed through observable clinical work rather than episodic exposure to rapidly outdated content. As an example of implementation, AI-related competencies aligned with this framework have been developed and approved through institutional curricular governance and are provided in Multimedia Appendix 1.

Nutrition provides a useful parallel example [4]. When framed as a CPC rather than an EPO, nutrition functions as a longitudinal capability integrated into clinical reasoning, patient counseling, interprofessional care, and prevention across the curriculum. For example, at our institution, nutrition-related competencies are addressed across multiple phases of training, including foundational science, clinical skills instruction, and clerkship experiences, rather than being confined to a single course or objective. This distributed approach reflects the inherently cross-cutting nature of nutrition and illustrates the challenge of aligning it with any single EPO. Together, AI and nutrition demonstrate how CPCs allow schools to address fast-evolving practice domains without repeatedly restructuring high-level program objectives.

## Implications for Curricular Governance and Accreditation

CPCs are intended to simplify, not expand, curricular structures. They are designed to be limited in number, periodically reviewed, and integrated across existing curricular structures, thereby reducing the need for additive content and minimizing fragmentation. In practice, they would be developed and periodically updated by existing curricular leadership bodies, such as curriculum committees or educational oversight groups, using the same governance processes already in place for curriculum review. Rather than creating new content blocks or standalone requirements, CPCs would be mapped across existing courses and clerkships, guiding where and how emerging domains are addressed longitudinally. Importantly, CPCs do not bypass existing governance structures. Because they are explicitly linked to established EPOs, they remain anchored to the core aims of the program and subject to the same oversight processes. This allows institutions to adapt to evolving domains of practice while maintaining alignment with accreditation expectations and overall curricular coherence.

CPCs differ from routine content review in that they define explicit, program-level expectations for emerging domains of practice. While content review may introduce new topics into individual courses, it does not ensure coordinated, longitudinal development across the curriculum. CPCs provide a structured approach to integrating these domains over time, making expectations visible, assessable, and aligned with program objectives. For example, a CPC in AI would not require a new course, but would inform how AI-related capabilities are integrated into clinical skills training, diagnostic reasoning sessions, and clerkship experiences over time. In this way, CPCs organize existing curricular elements more coherently rather than adding new ones.

This approach reduces the need for repeated modification of EPOs while providing a clear and adaptable mechanism for incorporating emerging domains of practice. For accreditation, CPCs offer a transparent way to demonstrate how programs respond to evolving clinical expectations without relying on continual expansion of high-level objectives or fragmented topic-based additions.

## Conclusion

The retirement or reorganization of “societal problems” should not be treated as a bureaucratic deletion. It can be interpreted as an opening to modernize how medical education defines and governs competencies. In a world where the pace of practice evolution is accelerating, curricula cannot be managed solely through reactive topic lists or through repeated expansion of high-level program objectives [6].

CPCs offer one such approach, translating enduring educational aims into the evolving realities of clinical work. They invite medical education to move from fixing yesterday’s problems to preparing for tomorrow’s practice intentionally, transparently, and with governance structures designed to keep pace. These challenges are especially acute in technology-mediated care environments.

## Appendix

Multimedia Appendix 1 Weill Cornell Medicine contemporary practice competencies for artificial intelligence.

## Abbreviations

AI: artificial intelligence
CPCs: contemporary practice competencies
EPA: entrustable professional activity
EPO: education program objective
LCME: Liaison Committee on Medical Education
